# Pulsed photo-ionization spectroscopy of traps in as-grown and neutron irradiated ammonothermally synthesized GaN

**DOI:** 10.1038/s41598-018-38138-6

**Published:** 2019-02-06

**Authors:** E. Gaubas, T. Čeponis, D. Meškauskaite, J. Mickevičius, J. Pavlov, V. Rumbauskas, R. Grigonis, M. Zajac, R. Kucharski

**Affiliations:** 1 Institute of Photonics and Nanotechnology, Vilnius University, Sauletekio av. 3, LT-10257 Vilnius, Lithuania; 2 Laser centre, Vilnius University, Sauletekio av. 10, LT-10222 Vilnius, Lithuania; 3 Ammono-Lab, Institute of High Pressure Physics, Sokolowska 29/37, 01-142 Warsaw, Poland

## Abstract

GaN-based structures are promising for production of radiation detectors and high-voltage high-frequency devices. Particle detectors made of GaN are beneficial as devices simultaneously generating of the optical and electrical signals. Photon-electron coupling cross-section is a parameter which relates radiation absorption and emission characteristics. On the other hand, photon-electron coupling cross-section together with photo-ionization energy are fingerprints of deep centres in material. In this work, the wafer fragments of the GaN grown by ammonothermal (AT) technology are studied to reveal the dominant defects introduced by growth procedures and reactor neutron irradiations in a wide range, 10^12^–10^16^ cm^−2^, of fluences. Several defects in the as-grown and irradiated material have been revealed by using the pulsed photo-ionization spectroscopy (PPIS) technique. The PPIS measurements were performed by combining femtosecond (40 fs) and nanosecond (4 ns) laser pulses emitted by optical parametric oscillators (OPO) to clarify the role of electron-phonon coupling. Variations of the operational characteristics of the tentative sensors, made of the AT GaN doped with Mg and Mn, under radiation damage by reactor neutrons have been considered.

## Introduction

GaN is prospective to become the next semiconductor generation for power electronics, for particle detectors and for other applications enabling much higher efficiency than silicon^1–3^. Ionizing radiation sensors made of GaN manifest optical and electrical signals, and these devices may operate as double response detectors. The ammonothermal (AT) growth method is one of the most promising technique, which allows the mass production of bulk GaN single crystals with low densities of structural defects^4^. However, crystals grown using the ammonothermal method contain a lot of impurities and other point defects. Some of them might be beneficial in formation of scintillation centres. Several impurities, such as Mg, Mn and carbon are intentionally introduced into GaN to fabricate the semi-insulating material featuring a small leakage current. Nontheless, introduction of the compesating impurities as usually leads to a formation of the fast recombination centres. The AT GaN can also be used as a seed material in growth of high quality GaN, using a hydride vapour phase epitaxy (HVPE), for fabrication of future devices based on electronic-grade GaN. The AT GaN grown showed the rather long carrier lifetimes^5,6^ and high radiation hardness^7^. A photon-electron coupling cross-section is the parameter which relates the radiation absorption and emission characteristics. The photon-electron coupling cross-section and photo-ionization energy, ascribed to the definite defect, represent fingerprints of deep centres in material. The efficiency of the conversion from the ionizing radiation or light absorption into luminescence may also depend on the electron-phonon coupling. The latter processes are sensitive to the excess carrier and phonon production rates. However, there still remains a lack of the detailed studies of parameters of the photon-electron and electron-phonon coupling ascribed to various defects and impurities in the as-grown and heavily irradiated AT GaN material. The defects usually affect the device operational characteristics by increasing the leakage current and reducing the charge collection efficiency (CCE)^8,9^. An identification of trap levels comprises the paramount importance for the future developments of GaN based devices for ionizing radiation detection^3^ and other applications.

In this work, the pulsed photo-ionization spectroscopy (PPIS) has been applied and combined with time-resolved photoluminescence (TR-PL) spectroscopy to trace the prevailing carrier traps. These techniques also allow to simultaneously control variations of carrier lifetime in contactless mode. The tentative capacitor and Schottky diode structures were made of AT GaN doped with Mg and Mn impurities. Variations of the detector operational characteristics dependent on the reactor neutron irradiation fluence have been examined. The femtosecond and nanosecond pulsed excitation has been combined to clarify the role of electron-phonon coupling in photo-ionization processes, attributed to different defects.

## Methods

The semi-insulating AT^10,11^ GaN crystals were grown by ammonothermal method in basic environment^11^. The supercritical ammonia is there applied to dissolve, to transport via convection and to crystallize GaN on native seeds. The AT technology is implemented using temperature range 400–600 °C and pressure of 1–4 kbar. Other parameters of the AT GaN material growth are described elsewhere^11^. The pristine material samples were rather highly doped with Mg (~2 × 10^18^ cm^−3^) atoms (GaN:Mg) and Mn (~10^19^ cm^−3^) dopants (GaN:Mn). The impurity spectrum and dopant concentration values were estimated by secondary ion mass spectroscopy (SIMS)^11^ and validated by electron spin resonance (ESR) measurements^7^. Several AT GaN samples were irradiated by nuclear reactor neutrons at Jožef Stefan Institute (Ljubljana) TRIGA reactor using a wide range of fluences *Φ* (10^12^–10^16^ cm^−2^).

**Figure 1:**
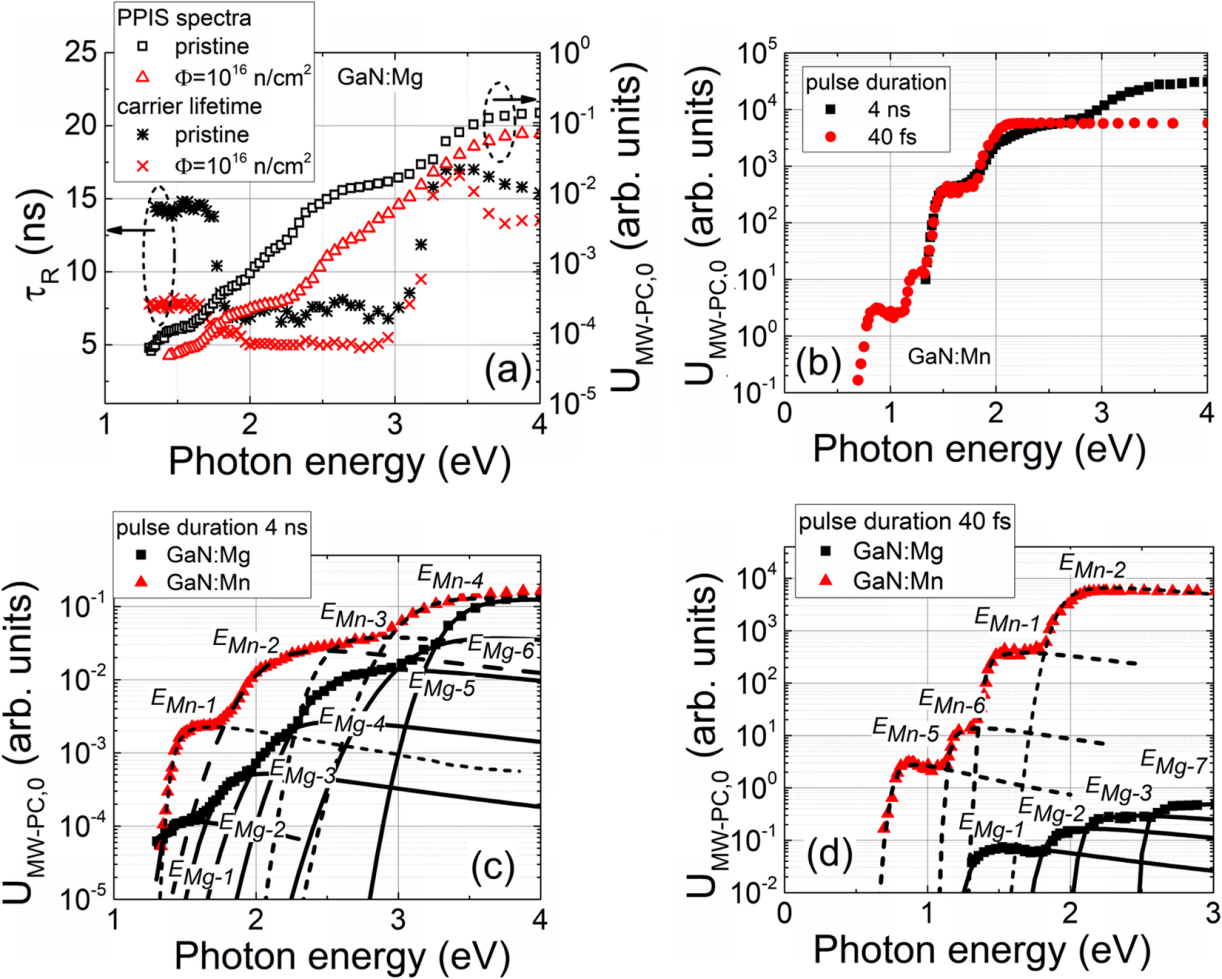
(**a**) Variations of the carrier recombination lifetime and MW-PC peak values as a function of photon energy, obtained for a fixed photon amount, in pristine and neutron irradiated (with fluence of *Φ* = 10^16^ n/cm^2^) GaN:Mg samples, measured using 4 ns excitation pulses. (**b**) Comparison of the PPIS spectra recorded on a pristine AT−grown GaN:Mn sample by using tuneable wavelength excitation pulses of 4 ns and 40 fs duration. Fitting (using Eq. (1)) of the PPIS spectra, recorded on pristine materials using excitation pulses of either 4 ns or 40 fs are depicted in figures (**c**) and (**d**), respectively. Symbols show the experimental data and short dash as well as solid curves represent simulated spectral steps for GaN:Mn and GaN:Mg, respectively.

The pulsed photo-ionization spectroscopy^12^ is beneficial relative to the direct photo-current technique as the dark leakage current is simply rejected in PPIS by a capacitive filter. Moreover, the changes of the recombination and trapping lifetimes of the photo-excited carriers are correlated with definite photo-ionization spectral steps and simultaneously controlled. Complementarily, the PPIS is recorded in contactless mode at room temperature, by excluding contact related effects. In this work, the PPI spectroscopy was performed using excitation by femtosecond (fs) and nanosecond (ns) lasers equipped with optical parametric oscillators (OPO). A nanosecond OPO instrument Ekspla NT342B with pulse duration of 4 ns as well as wavelength tuning range from 210 to 2300 nm, and a Ti:sapphire laser based OPO system with pulse duration of ~40 fs as well as wavelength range of 350–2500 nm were employed. The peak values (*U*_*MW-PC,0*_) in the microwave probed photoconductivity (MW-PC) transients were recorded to estimate the excess carrier density generated by the photo-ionization processes. The MW-PC signal *U*_*MW-PC*_ is there proportional to a density of the photo-excited carriers, while their relaxation rate within a transient represents the carrier lifetime ascribed to later stages of trap filling/emptying. The sample was placed on a slit-antenna of the 21–22 GHz microwave (MW) system and excited by OPO laser beam, starting from long wavelength wing, to avoid simultaneous filling of several traps. The transients of the MW-PC response were recorded on 50 Ohm load resistor connected in series with MW detector, by using a 2 GHz oscilloscope LeCroy Wave Runner 620Zi. Variations of the *U*_*MW−PC,0*_ values dependent on the incident photon energy represent the step-like photo-ionization spectra (Figure 1(a)). The simultaneous changes of the excess carrier decay lifetime are obtained by measuring a time interval needed for *U*_*MW-PC*_ reduction to a *U*_*MW-PC,0*_ × e^−1^ value. The MW-PC transient shape also provides an additional information concerning the dominant and competing carrier decay processes.

The time resolved photoluminescence (TR-PL) measurements were carried out by a Streak Camera (SC) technique. A Hamamatsu C10627 streak-camera along with an Acton 2300 spectrometer were used for the measurements of PL spectra and transients. The SC technique provides the temporal and spectral evolution of the PL intensity under high-level excitation by a short laser pulse. These TR-PL measurements were performed using the 290 fs duration PHAROS laser pulses at 315 nm wavelength (generated by optical parametric oscillator ORPHEUS of 100 μW power and 10 kHz repetition rate).

The sensor functional characteristics were tested by recording the current transients. The tentative capacitor^7^ and Schottky type detectors were made of the c-oriented GaN:Mg and GaN:Mn semi-insulating materials. Metallization, to get ohmic and Schottky barriers, was implemented by vacuum evaporation procedures using electron beams. The ohmic contacts were fabricated using procedures of the Ti/Al/Ni/Au (30/90/20/100 nm) metal thin-film deposition, followed by the rapid thermal annealing (RTA), similarly to the procedures referenced in ref.^13^. The Schottky contacts were made of Ni/Au (25/200 nm) metal stack. Each detector contained a narrow hole opening within metallization layer, to implement an optical injection of surface domain of the excess carrier pairs. The transients of the injected charge drift current^14^ (ICDC) were recorded on 50 Ω load at applied voltage of ~300 V by using a 2 GHz oscilloscope LeCroy Wave Runner 620Zi.

## Results and Discussion

Variations of the carrier recombination lifetime as a function of photon energy (when using 4 ns pulses) and the PPIS spectra recorded on pristine and neutron irradiated by *Φ* = 10^16^ n/cm^2^ GaN:Mg samples are illustrated in Fig. 1(a). The photon-electron coupling with *n*_*d0*_ trapped carriers (on a definite energy *E*_d_ level characterized by the *σ*_*d*_*(hν*) interaction cross-section) determines the spectral changes of an absorption coefficient *α(hν*) = *σ*_*d*_*(hν*)*n*_*d0*_. The density of photo-emitted carriers n^*^_d_ = *σ*_*d*_*(hν*)*n*_*d0*_*F(hν*) determines the peak value (*U*_*MW−PC,0*_) ~ n^*^_d_ ~ *σ*_*d*_*(hν*) of the *U*_*MW-PC*_ for a fixed surface density *F(hν*) = *const* of the incident photons of varied energy. The *F(hν*) is evaluated by calibration of the energy per pulse measurements within incident laser beams.

The PPIS steps, recorded as *U*_*MW-PC,0*_*(hν*) ~ *σ(hν*) spectral distribution, represent the ratios of *σ(hν*) ascribed to different (*L*) deep centres, at *F(hν*) = const. The shape (*σ*_*d*_*(hν*)) and spectral position (*E*_d,L_) of the *σ*_*dL*_*(hν*) ~ *U*_*MW-PC,0*_ steps serve for evaluation of the photo-activation energy *E*_d,L_ and identification of definite (*L*) defects. As usually, several traps^12^ appear to be active, and PPIS spectrum contains a few spectral steps. The relative concentrations *N*_*d,L*_ of different defects *L* can be evaluated by using a spectrum (e.g. UV-VIS range transmission) of an absorption coefficient *α(hν*), independently measured on the same sample, and its correlation with *σ(hν*) ~ *U*_*MW-PC,0*_, as: *N*_*d,L*_(*hν)* = *α(hν)*/*σ*_*dL*_*(hν)*. The absolute values of *N*_*d*_ → *N*_*T*_ are deduced by identifying relative values of *N*_*d,L*_ (re-plotting the *N*_*d*_(*hν)* scale) with known concentration of centres (for instance, Mg, Mn dopants) independently obtained for the same sample (e.g. from SIMS^11^, ESR measurements^7^). The scale of the absolute values of *σ(hν*) can then be obtained (by shifting an arbitrary scale to values of *σ*_*h*_*(hν*) = *α*_*h*_*(hν)/N*_*h*_). This re-scaling is performed by using *α*_*h*_ values, obtained from UV-VIS transmission measurements at homogeneous excitation *α*_*h*_*w* ≪ 1, where thickness *w* is of about 400 μm for the employed samples.

The electron-phonon coupling can play the important role in formation of PPIS steps, determined by a photon-electron coupling. Different methods, those include the electron-phonon coupling, had been developed^15–20^ to fit the absorption peak onset. The phonon-assisted changes of the cross-section *σ(hν*) for a definite defect can be approximated by the Kopylov- Pikhtin^20^ approach

1 $$\sigma (h\nu )\propto {\int }_{0}^{\infty }\frac{{e}^{-{(E+{E}_{d}-h\nu )}^{2}/{{\rm{\Gamma }}}^{2}}\sqrt{E}dE}{h\nu {(E+{E}_{d})}^{2}},$$

where electron-phonon coupling is determined by the broadening parameter *Γ*. The broadening of the absorption onset is also related to the Huang-Rhys^21^ factor and, consequently, to the Franck-Condon shift and the energy of the vibrational mode^22^. It can be deduced from simulations, using Eq. (1), that the value of the photon-electron coupling cross-section increases with shallowing (*E*_*d*_) of levels. The cross-section of the photon-electron coupling directly determines the efficiency of the conversion of absorption into emission^23^.

The Kopylov-Pikhtin^20^ approach (Eq. (1)) has been applied for simulating *U*_*MW-PC,0*_*(hν*) of the experimental spectra by varying *E*_*d*_ and *Γ* as free parameters to get the best fit estimated by a non-linear least square method. The carrier lifetime *τ*_*R*_ variations correlated with excitation wavelength changes have been monitored by analysing the *U*_*MW-PC*_(*t*) transients. Carrier lifetime variations, depicted in Fig. 1(a) for the pristine GaN:Mg, show that nearly constant carrier lifetime is associated with the shallowest PPI spectral step. However, it decreases and retains the shortened *τ*_*R*_ values in the range of moderate excitation photon energies used. The *τ*_*R*_ increases and shows non-monotonous variation in the range of the largest photon energies employed (Fig. 1(a)). The *τ*_*R*_ variations can be explained by the processes of the photo-neutralization and photo-ionization of charged traps.

The excitation pulses of 40 fs and 4 ns durations were used to envisage an impact of the electron-phonon coupling. The 40 fs excitation pulses seem to be shorter than the energy relaxation time, and a photo-ionization process runs within nearly adiabatic regime. For 4 ns pulses, the MW-PC response represents the process integrated over energy relaxation times. Qualitatively, a shift of the position of the peak of a PPI step, and the steepness of the spectral-step slope should appear relative to the pronounced electron-phonon coupling, in the case of short excitation pulses. The PPI spectra obtained for GaN:Mn by using different excitation pulses, are compared in Fig. 1(b). The same dominant spectral steps (on pristine GaN:Mn sample) can be deduced from Fig. 1(b) for the definite ranges of a photon energy variation. There spectral steps, obtained for different excitation pulse durations, qualitatively correlate. The mostly pronounced difference in steepness of the slope has been observed for the spectral step with a peak nearby 2 eV. This implies the strongest electron-phonon coupling ascribed to this defect where the lower steepness for 4 ns pulses is obtained. For quantitative characterization of the electron-phonon coupling, the *Γ* factors have been evaluated by fitting (using Eq. (1)) the shape and spectral position of a peak of each spectral step within measured spectrum, where *E*_*d*_ together with *Γ* are free variables.

Four trap levels have been revealed for the GaN:Mn samples and six traps have been separated for the GaN:Mg samples by fitting the experimental spectra recorded for the excitation pulses of 4 ns duration (Fig. 1(c)). The activation energies (with fitting uncertainties of about 5%) and *Γ* parameters for the predominant centres have been identified, as listed in Table 1. The defects (Table 1) have tentatively been identified using the defect activation energy values taken from literature.

**Table 1:** Values of the photo-activation energy and of broadening parameter *Γ* extracted from fitting of the PPIS peaks recorded by using the 40 fs (denoted by a superscript fs) and 4 ns (denoted without any superscript) excitation pulses in the pristine and irradiated (denoted by a superscript irr). The assignment of defect type with definite activation energy has been performed by comparing with literature data referenced.

Pristine		Irradiated with *Φ* = 10^16^ cm^-2^		
**GaN:Mn**				
Photo-activation energy (eV) ±0.02 eV	*Γ*^*ns*^/*Γ*^*fs*^	Photo-activation energy (eV) ±0.04 eV	*Γ*^*ns*^/*Γ*^*fs*^	Defect type
*E*^*fs*^_*Mn-5*_ = 0.75	*−*/0.05			Unidentified
*E*^*fs*^_*Mn-6*_ = 1.14	*−*/0.03	*E*^*fs*^_*Mn-6*_^*irr*^ = 1.09	*−*/0.11	Unidentified
*E*_*Mn-1*_/*E*^*fs*^_*Mn-1*_ = 1.40/1.39	0.05/0.04	*E*_*Mn-1*_^*irr*^/*E*^*fs*^_*Mn-1*_^*irr*^ = 1.42/1.41	0.08/0.03	Mn related^25^
*E*_*Mn-2*_/*E*^*fs*^_*Mn-2*_ = 1.98/1.90	0.25/0.08	*E*_*Mn-2*_^*irr*^/*E*^*fs*^_*Mn-2*_^*irr*^ = 1.98/1.91	0.22/0.08	Mn related^26^
*E*_*Mn-3*_ = 2.40	0.15/*−*	*E*_*Mn-3*_^*irr*^ = 2.39	0.25/*−*	Interstitial^27^ Ga_I_
*E*_*Mn-4*_ = 2.97	0.25/*−*	*E*_*Mn-4*_^*irr*^ = 2.96	0.28/*−*	Unidentified^27^
**GaN:Mg**				
*E*_*Mg-1*_ /*E*^*fs*^_*Mg-1*_ = 1.30/1.27	0.02/0.08			Donor^28^ or C_I_ acceptor^29,30^ state
*E*_*Mg-2*_/*E*^*fs*^_*Mg-2*_ = 1.75/1.78	0.18/0.07	*E*_*Mg-2*_^*irr*^ = 1.75	0.23/*−*	Mg related^31^
*E*_*Mg-3*_/*E*^*fs*^_*Mg-3*_ = 2.07/2.06	0.23/0.05	*E*_*Mg-3*_^*irr*^ = 2.05	0.27/*−*	Vacancy^32^ V_Ga_
*E*_*Mg-4*_ = 2.39	0.15/*−*	*E*_*Mg-4*_^*irr*^ = 2.38	0.25/*−*	Interstitial^27^ Ga_I_
*E*^*fs*^_*Mg-7*_ = 2.50	*−*/0.03	*E*_*Mg-7*_^*irr*^ = 2.45	0.16/*−*	Vacancy^30^ V_Ga_
*E*_*Mg-5*_ = 3.10	0.32/*−*	*E*_*Mg-5*_^*irr*^ = 3.10	0.35/*−*	Mg related^31^
*E*_*Mg-6*_ = 3.30	0.2/*−*	*E*_*Mg-6*_^*irr*^ = 3.30	0.27/*−*	Vacancy^27^ V_N_

The OPO laser system with 40 fs excitation pulses provided the slightly narrower range of excitation wavelengths within a UV spectral wing than that of 4 ns OPO system. Additionally, the increased sensitivity of PPIS using 40 fs pulses allowed registering of the shallower traps. Therefore, the spectral steps for only a few same levels (Fig. 1(b–d)) can be compared in PPIS recorded by using 4 ns and 40 fs excitation regimes.

For 40 fs excitation pulses (Fig. 1(d)), the PPIS on GaN:Mn exhibited the additional peaks *E*_*Mn-5*_ and *E*_*Mn-*6_ together with Mn impurities ascribed levels *E*_*Mn-1*_ and *E*_*Mn-2*_, observed in PPIS recorded using 4 ns excitation pulses. The photo-activation energies of these additional centres have been extracted as *E*_*Mn-*5_ = 0.75 eV, *E*_*Mn-6*_ = 1.14 eV by fitting the experimental data. The origin of these deep levels with photo-activation energies of *E*_*Mn-5*_ = 0.75 eV and of *E*_*Mn-6*_ = 1.14 eV had not been reported in literature. In Mg doped pristine GaN samples, an additional peak *E*_*Mg-7*_ = 2.50 eV has been deduced (by fitting the spectra recorded for 40 fs pulses) together with those traps *E*_*Mg-*1_, *E*_*Mg-2*_ and *E*_*Mg-3*_, revealed by measuring PPIS for 4 ns pulses. The parameters extracted from fittings of the PPI spectral steps, recorded using 40 fs pulses, are also listed in Table 1.

Introduction of defects by neutron irradiations does not change considerably a structure of the photo-ionization spectra (Fig. 1(a)), where the same PPIS peaks, inherent for specific materials, can be resolved. Values of fitting parameters obtained for 4 ns and 40 fs excitation pulses in pristine and irradiated samples are listed in Table 1. As an exception, the deep centre with energy of *E*_*Mg-7*_^*irr*^ = 2.45 eV can additionally be separated in the PPIS spectrum recorded for the irradiated GaN:Mg samples. The main difference appears in density of photo-active centres, which can be deduced from variations of the *U*_*MW−PC,0*_, when comparing PPIS obtained for the pristine and 10^16^ n/cm^2^ neutron fluence irradiated samples.

Such the observations can be explained by a rather small concentration (≤10^16^ cm^−3^) of the introduced radiation defects in comparison with intrinsic defect and dopant^7^ densities (>10^18^ cm^−3^). The type of the resolved traps (relative to their charge-state) seems to be invariable after neutron irradiations, as a profile of carrier lifetime variations retains (Fig. 1(a)). Nevertheless, carrier lifetime values are significantly reduced for irradiated samples. This indicates that radiation defects act mostly as the carrier non-radiative recombination centres.

It can be deduced from Table 1 that the electron-phonon coupling characterized by the factor *Γ* retains (within errors of measurements and fitting) for the most of the resolved PPIS peaks when comparing the pristine and irradiated material samples. However, the *Γ* parameter is obtained to be significantly different when comparing the PPIS recorded using 4 ns and 40 fs excitation pulses. In the latter case of 40 fs pulse durations, values of *Γ* factor do not exceed 0.11 in all the examined samples, indicating a rather weak electron-phonon coupling even at room temperature.

**Figure 2:**
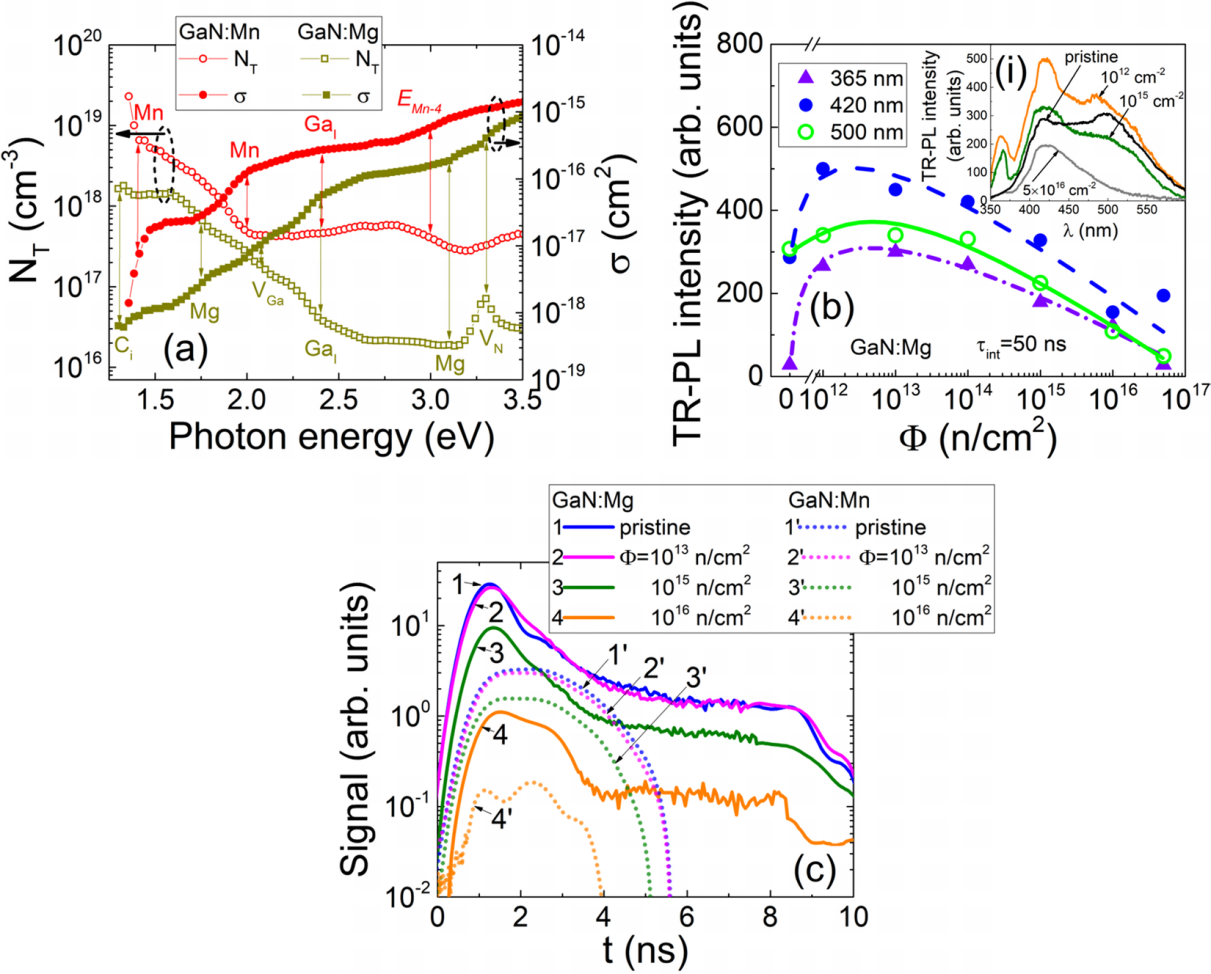
(**a**) Variations of cross-sections of the photon-electron coupling and of trap concentration as a function of excitation photon energy in the pristine AT-grown GaN samples doped with Mg and Mn. Vertical arrows indicate the peak positions of the PPIS steps, ascribed to different centres. (**b**) Variations of the predominant TR-PL spectral peaks with neutron irradiation fluence. In the inset (*i*) variations of TR-PL spectra are depicted. (**c**) Variations of the current transients (in the tentative detectors made of GaN:Mg and GaN:Mn for the same test excitation parameters) dependent on neutron irradiation fluence.

The cross-sections of the electron-photon coupling and the concentrations of defects have been estimated by combining data of the independent measurements. There, values of the absorption coefficient were measured on each sample by using UV-VIS transmission spectroscopy^7^. The quantity of absorbed photons was calibrated using the transmitted beam energy measurements. Concentration of the main dopants was calibrated using the SIMS^11^ as well as ESR data^7^ and by relating them to the MW-PC peak values. The spectral variations of the cross-sections (*σ*) ascribed to several species of traps and trap densities (*N*_*T*_) are depicted in Fig. 2(a) for GaN:Mg (a) and GaN:Mn (b) pristine materials, respectively.

The double-response detectors of the ionizing-radiation or high energy particles operate through generation of secondary carrier pairs. These carrier pairs drift, due to electric field within inter-electrode gap of the junction or capacitor-like structures^24^, causing the current signals (Fig. 2(c)). These carriers also recombine through radiative recombination centres, producing scintillation signals (Fig. 2(b)). Such an operation mode can be applied for both the tracking of particles^24^ and the spectroscopy of energy of the incident particles (owing to proportionality of the collected secondary pair charge) during irradiation. These GaN sensors are nearly blind to the environmental light and manifest rather small dark/leakage currents. Also, GaN sensors are able to operate as dosimeters of the large fluence irradiations exhibiting the changes of both the luminescence intensity (Fig. 2(b)) and electrical signal (recorded either on electrodes (Fig. 2(c)) or in contactless mode, by measuring the microwave response) due to radiation defects.

An increase of concentration of the neutron introduced radiation defects was more clearly deduced from UV-VIS transmission spectra^7^. These radiation defects act mostly as carrier radiationless recombination centres. Consequently, the radiation defects lead to a decrease of the TR-PL intensity or even disappear of a few of scintillation spectral peaks (Fig. 2(b)). Thereby, the non-radiative recombination through radiation defects suppresses the efficiency of absorption conversion to radiation in double-response GaN detectors. The current signals, tested at the same excess carrier pair injection parameters in tentative GaN sensors (Fig. 2(c)), also show a reduction of the electrical response with increase of neutron irradiation fluence. However, value of current and duration of current pulses significantly depend on the pristine material. The longer ICDC transients and the larger pulsed currents have been obtained for detectors made of GaN:Mg material (which showed the longer excess carrier lifetimes in the pristine samples^7^) than that of GaN:Mn. This clearly indicates the rather large densities of radiationless defects appeared in formation of the semi-insulating GaN material where compensating impurities simultaneously act as the non-radiative recombination centres. While, radiation damage enhances density of non-radiative carrier capture centres by suppressing the electrical and scintillation signals in ionizing radiation detectors.

In summary, the pulsed photo-ionization spectroscopy (PPIS) on ammonothermal GaN, heavily doped with Mg and Mn, has been performed in contactless MW-PC mode by using femtosecond and nanosecond laser pulses of tuneable wavelength in the range of 210–2500 nm. The activation energy *E*_*d*_ and broadening factor *Γ* parameters have been evaluated for several PPI spectral steps. It has been revealed a reduction of the broadening factor *Γ* for short 40 fs excitation pulses relative to those *Γ* values estimated for 4 ns pulses. This result implies the weakened electron-phonon coupling when energy relaxation time for excess carriers is longer than a laser pulse duration. In the PPIS recorded on GaN:Mg, seven traps have been resolved, associated with vacancies, interstitials, impurities and dopants. In the PPIS recorded on GaN:Mn, a set of five traps has been resolved. A profile of carrier lifetime variations illustrated for GaN:Mg implies that the PPIS steps associated with *E*_*Mg-1*_ and *E*_*Mg-6*_ defects can be associated with photo-neutralization processes, while other *E*_*Mg-2*_–*E*_*Mg-6*_ PPIS peaks represent the photo-ionization processes. The tentative double response detectors of ionizing radiation made of the semi-insulating AT GaN material exhibited functionality even under neutron irradiations with fluences up to 10^16^ cm^−2^. However, the efficiency of conversion of the carrier pair production by the incident radiations into scintillation and current signals significantly drops with increase of accumulated radiation fluence.

## Data Availability

All data generated or analysed during this study included in this published article are available from the corresponding author on reasonable request.
